# Giant phagocytes (Gφ) and neutrophil-macrophage hybrids in human carotid atherosclerotic plaques – An activated phenotype

**DOI:** 10.3389/fimmu.2023.1101569

**Published:** 2023-02-24

**Authors:** Lena Lavie, Erez Si-on, Aaron Hoffman

**Affiliations:** ^1^ Unit of Anatomy and Cell Biology, The Ruth and Bruce Rappaport Faculty of Medicine, Technion-Israel Institute of Technology, Haifa, Israel; ^2^ Department of Vascular Surgery and Transplantation, Rambam Health Care Campus, The Ruth and Bruce Rappaport Faculty of Medicine, Technion-Israel Institute of Technology, Haifa, Israel

**Keywords:** human carotid plaques, neutrophil-derived giant phagocytes (Gφ), neutrophil-macrophage hybrids, foam cells, CD66b, CD163, CD68, atherosclerosis

## Abstract

**Introduction:**

A small subpopulation of CD66b+ neutrophils with extended lifespan and immensely large size was identified *in vitro*. They internalized dead neutrophil remnants and cellular debris, transforming them into giant phagocytes (Gφ) resembling macrophage-foam cells with a massive lipid content and CD68+ scavenger receptor expression. Thus, we sought to investigate if similar CD66b+ neutrophils with altered morphology and functions exist in inflammatory/atherosclerotic conditions *in vivo*, by using human carotid atherosclerotic plaques.

**Methods:**

Thirty-three plaques were obtained from 31 patients undergoing endarterectomy. Carotid plaques were analyzed for CD markers by immunohistochemistry and immunofluorescence and quantitatively analyzed by confocal microscopy. Intra-plaque lipids were stained with Oil Red O.

**Results:**

Plaque CD66b+ neutrophils co-expressed myeloperoxidase (MPO)+ and neutrophil elastase (NE)+. Also, co-expression of CD66b+/CD68+, CD66b+/CD36+, CD66b+/vascular-endothelial-growth- factor (VEGF)+ and 3-nitrotyrosine (3-NT)+/NE+ was noted. Similarly, macrophages co-expressed CD163+/CD68+, CD163+/VEGF+ and CD163+/3-NT+. Both cell types were predominantly localized in lipid-rich areas and stained for lipids. CD66b+ and CD163+ expressions were highly positively correlated with each other and each with CD68+, and 3-NT+. Morphologically, CD66+ cells were big, had a rounded nucleus, and resembled macrophage-foam cell morphology as well as that of Gφ *in vitro*. To clarify whether CD66b+ and CD163+ cells represent two distinct plaque-populations, plaques were double-stained for CD66b/CD163 co-localization. A third of the plaques was negative for CD66b/CD163 co-localization. Other plaques had a low co-localization, but in few plaques, co-localization was high, collectively, indicating that in some of plaques there were two distinct cell populations, those resembling Gφ, and those co-expressing CD66b+/CD163+, demonstrating a hybrid neutrophil-macrophage phenotype. Interestingly, CD66b+/CD163+ co-localization was highly positively correlated with the oxidant 3-NT, hence, supporting trans-differentiation of CD66b+ cells to CD163+ Cells. Conversely, phagocytosis of dead neutrophils by macrophages might have also occurred.

**Discussion:**

Thus, we conclude that in some of the plaques CD66b+ cells might represent cells resembling Gφ that developed in prolonged culture conditions. Yet, CD66b+/CD163+ co-expressing cells represent a new neutrophil-macrophage hybrid population of unknown transitioning point, possibly by adopting macrophage markers or contrariwise. Nonetheless, the significance and functions of these cells in plaque biology or other inflammatory/atherosclerotic conditions should be unveiled.

## Introduction

Typically, neutrophils were viewed as a homogenous short-lived population of terminally differentiated cells with limited transcriptional capacities, possessing specific killing abilities against invading pathogens. In recent years however, mounting evidence demonstrates that neutrophils are a heterogenous group of cells, presenting vast phenotypic and functional heterogeneity (1). Along this line, we described for the first time a new and novel subpopulation of blood derived neutrophils which spontaneously develop in prolonged culture conditions (2–4). Freshly isolated blood neutrophils in culture are known to have a very short lifespan among leukocytes and are constitutively committed to apoptosis. Notably, in culture, most neutrophils become apoptotic within 24 hrs (5, 6). Yet, by maintaining neutrophils in prolonged culture conditions, without additional growth factors or cytokines, a small number of Annexin-V negative/LC3B+ neutrophils remain viable and display an extended life span. Within 3 days they become immensely vacuolated, acquiring an excessively large size by internalizing granules, cellular residues and debris of non-viable neutrophils, thus, becoming giant phagocytes (Gφ) (2, 3). Moreover, unlike other types of giant phagocytic cells which are multinucleated heterokarions, arising from cell-cell fusion, these neutrophil-derived Gφ have a single nucleus (7). They also differ from monocytic macrophages by their loose attachment to the surface of the tissue culture dish and their movement abilities in culture differ from those of macrophages, as demonstrated by time lapse-photography (2). In addition to these unique morphological features, these cells are devoid of monocytic (CD14, CD163, CD16) or dendritic (CD1c, CD141) cells markers while exhibiting phenotypic markers and functions of neutrophils. Their neutrophilic origin is evident by the expression of the specific neutrophil granules marker - CD66b, azurophilic granules markers - CD63, and myeloperoxidase (MPO). Also, Gφ express additional neutrophilic markers including neutrophil elastase (NE), CD11b and CD15. Functionally, these Gφ take-up latex beads and opsonized zymosan, express the NADPH oxidase gp91-*phox* and p22-*phox* subunits and generate reactive oxygen species (ROS) in response to stimulation with PMA (2–4). However, unlike freshly harvested neutrophils, Gφ also intensively express the scavenger receptors CD68 and CD36, and take-up oxidized low density lipoprotein (oxLDL). An additional unique feature of Gφ in culture is their ability to generate ROS in response to stimulation with oxLDL. The presence of LC3B-coated vacuoles and LC3B aggregates in Gφ indicate that autophagy is a constitutive trait in these cells.

Treating Gφ with various inhibitors affected Gφ development in culture. Inhibiting NADPH oxidase by diphenyl iodide (DPI), ROS production by N-acetylcysteine (NAC), phagocytosis by cytochalasin B, or inhibiting PI3K pathway - all abolished their formation in culture. Additionally, inhibiting autophagy or culturing neutrophils in the presence of GM-CSF/IL-4 also abolished their development. These latter findings indicate that functional phagocytosis, NADPH-oxidase, ROS signaling as well as autophagy are essential for their development in culture conditions (2, 4).

However, based on their phenotype and functions studied thus far, it is unclear whether neutrophil-derived Gφ bear any significant relevance to physiological or pathological conditions *in vivo*.

The human carotid plaque represents an *in vivo* easily attainable atherosclerotic tissue which enables to analyze the composition of inflammatory cells in human arterial disease. In earlier studies, most cellular analyses of carotid plaques primarily focused on macrophage foam cells and T cells. However, in the last decade the contribution of neutrophils to atherosclerotic plaque destabilization and rupture emerged as an important factor (8, 9). In a comprehensive study by Ionita et al. high neutrophil numbers were associated with a large lipid core in human atherosclerotic carotid plaques and more so in symptomatic patients. These increases in neutrophil numbers were also associated with histopathologic features of rupture-prone atherosclerotic lesions, suggesting plaque destabilization (10). Hosokawa et al. identified infiltration of esterase activity and CD66b+ neutrophils in seven human atherosclerotic carotid plaques which appeared morphologically activated and expressed oxidant activity (11). Of note, Naruko et al. have shown that neutrophil infiltration in plaques was actively associated with acute coronary events. Moreover, all patients who had died from acute myocardial infarction (AMI) had neutrophils within their plaques, whereas in coronary lesions of patients who had died of non-cardiovascular disease, neutrophils were extremely rare (12).

In the current study we sought to investigate whether this cell phenotype that spontaneously developed in culture conditions, might be present in human arterial atherosclerotic disease, such as the carotid plaques of patients undergoing endarterectomy, and might be associated with inflammatory/anti-inflammatory conditions *in vivo*. Neutrophils are difficult to study, particularly in tissues, due to their propensity for activation and trans-differentiation (13, 14). Nonetheless, the presence of cells resembling neutrophil-derived Gφ was confirmed based on their specific CD markers combined with their morphology. These cells, like Gφ in culture, expressed the specific neutrophil marker CD66b, neutrophil elastase (NE) myeloperoxidase (MPO) and CD63. Additional markers which were previously identified in Gφ in culture included CD68 and CD36 scavenger receptors, 3-nitrotyrosin (3-NT) and lipids. In parallel, because of their foam cell morphology monocytes/macrophages were also identified by their exclusive CD163 marker. Due to the morphological similarities between CD66b+ and CD163+ expressing cells, CD66b+/CD163+ co-localization experiments were performed. In several plaques CD66b/CD163 co-localization was not observed, in other plaques a low co-localization was noted, and in few plaques CD66b+/CD163+ co-localization was high. Thus, indicating the presence of several populations of CD66b/CD163 expressing cells in human carotid plaques.

## Materials and methods

### Patients

A total of 33 plaques were obtained from 31 patients (21 Males/10 Females) undergoing endarterectomy in the Department of Vascular Surgery and Transplantation, Rambam Medical Centre, using standard surgical techniques (15). Surgery was performed if carotid stenosis was 50% or more in clinically symptomatic patients, in those who had either ipsilateral cerebrovascular event (CVA) or transient ischemic attack (TIA) or amaurosis fugax in the last 180 days, and in clinically asymptomatic patients if the stenosis was 70% or higher. Asymptomatic patients included those with carotid artery stenosis without a history of ischemic stroke, TIA or other neurologic symptoms referable to the carotid arteries as defined by NASCET-North American Symptomatic carotid Endarterectomy Trial (16). Demographic data, comorbidities, risk factors, medications, and blood chemistry were recorded. The protocol was approved by The Local Humans Rights Committee of RAMBAM Medical Center (RMB-0175-14), according to the declaration of Helsinki, and all patients signed an informed consent form.

### Preparation of the specimens

Carotid plaques were removed by standard surgical techniques and minimal manipulation to the specimens. Immediately after the surgery, plaques were stored in phosphate buffered saline (PBS) at 4°C. The specimens were embedded into an optimum cutting temperature (OCT) compound (LEICA, 020108926) and stored at -80°C for further analysis. Samples were analyzed by immunohistology, immunohistochemistry, and immunofluorescence using confocal microscopy. For immunohistochemistry plaque samples were analyzed for various CD cellular markers including CD66b, CD163, CD68, and lipids. Additional cellular markers were used for confocal microscopy. Quantitative analyses of the expression of various markers were performed as previously described (2, 15) and as specified below. Mouse monoclonal primary antibodies were used to identify neutrophils (anti-CD66b), macrophages-foam cells (anti-CD163) and anti-3-nitrotyrosine for oxidative-nitrosative stress. Rabbit primary polyclonal antibodies were used for double-labeling the cells with additional markers including scavenger receptors anti-CD68 and anti-CD36, anti-NE, anti-MPO, anti-Vascular Endothelial Growth Factor (VEGF), anti- CD31, and anti- smooth muscle actin (SMC-actin). Polyclonal anti-CD163 was used for double-labeling with anti-CD66b. Intra/extra cellular lipids and lipid crystals were determined by immunohistochemistry with Oil Red O staining (15).

### Antibodies for immunohistochemistry and immunofluorescence

Mouse monoclonal primary antibodies included: anti-CD66b (80H3, AbD Serotec), anti-CD163 (GHI/61), anti-3 nitrotyrosine (3-NT), and anti-smooth muscle cell actin (SMC-actin) (Santa Cruz Biotechnologies, Inc. CA), HIF-1α (GT10211, Gen Tex). Rabbit primary polyclonal antibodies included: anti-CD68 (ProteinTech, USA), anti-CD36 (SR-B3, Novus), anti- myeloperoxidase (MPO) (ab45977, Abcam, UK), anti-neutrophil elastase (NE) (Calbiochem, San Diego, CA), anti-CD163 (M-96) (Sc-33560, Santa Cruz Biotechnologies, Inc. CA) anti-CD31 (ab32457, Abcam, UK), and VEGF (Abcam, UK). Secondary antibodies included: Cy2 (CF 488A)-conjugated goat anti-rabbit IgG and/or Cy5 (CF 647)-conjugated goat anti-mouse IgG (Biotium, Hayward, CA). Isotype controls included: purified mouse IgG1(clone MG1-45) and IgG2 (clone MOPC-173, BioLegend, San Diego, CA), and rabbit IgG (sc-2027, Santa Cruz Biotechnologies, Santa Cruz, CA).

### Immunohistochemistry

Frozen sections were cut at a 5 μm thickness and mounted on microscope slides. The 5-μm-thick sections were stained with hematoxylin and eosin (H&E), and lipid deposits in the plaques were visualized by Oil Red O staining as previously described (3, 15). Primary antibodies against CD66b, CD163, and CD68 cellular markets were diluted to 1:100, and a Histostain-Plus Kit AEC, Broad Spectrum (Invitrogen) was used for their detection. The sections were incubated with the primary antibodies for 2 hrs. at 37°C. Then, the sections were incubated with secondary antibody from the Histostain-Plus kit, for 30 min at 37°C. The 3-amino-9-ethylcarbazole (AEC) was used as a chromogen to detect the antibodies according to manufacturer’s instructions. Polymorphonuclear neutrophils (PMNs) were identified by the expression of CD66b, macrophages were identified by the expression of CD163 and foam cells were identified by the expression of CD68 scavenger receptors. Isotype controls were used as specified in the list of antibodies. Lipid deposits were stained with Oil Red O. Sequential sections were stained each for an antibody including for negative controls. At least 3 different sections were cut from the center of each plaque for each CD marker and in each section at least 5 different fields were analyzed.

Collagen and non-collagen proteins were detected by differential staining in tissue sections with two dyes - Sirius Red for all collagens and Fast Green for non-collagen proteins.

### Immunofluorescence and confocal microscopy

Frozen sections were cut at a thickness of 5 μm and mounted onto microscope slides. Sequential sections were used for a single antibody or for two antibodies (co-expression- as specified in **Tables 1**, **2**) including for the negative controls. Samples were fixed with acetone for 15 min. at 4°C and washed with PBS. Then, incubated with the primary antibodies diluted at 1:100 in blocking buffer of 10% normal goat serum in RPMI-1640 medium, overnight at 4°C (2). The following cellular markers were used to identify and quantify cell populations in the carotid plaques; PMNs were identified by primary antibodies for CD66b, NE, and MPO and macrophages were identified by CD163. Double staining of CD66b(mono)/CD163(poly) was performed to identify potential co-expression. Additional markers included the scavenger receptors CD36 and CD68 for foam cells, the oxidative stress marker 3-NT, hypoxia inducible factor 1α (HIF-1α), VEGF, CD31 – for vessel identification by the presence of endothelial cells, and smooth muscle cell actin (SMC-actin), a marker of arterial wall remodeling.

**Table 1:** Quantitative analysis of cellular carotid plaque markers (by % stained area ± SD) from the total plaque area and relatively to CD163+ and CD66b+ expressing cells.

Cellular Markers	N	% Stained Area	(*) Relative % To CD163+ (mono)	(#) Relative % To CD66b+ (mono)
Neutrophils, CD66b+ (red)	33	0.85 ± 1.69(#)	78.0 ± 155.0(*)	**100.0% **± **198.8(#)**
Macrophages, CD163+ (mono) red	32	1.09 ± 1.85(*)	**100% ± 169.7**	128.2 ± 217.6
Macrophages, CD163+ (poly) green	29	1.35 ± 1.50	123.9 ± 137.9	158.8 ± 176.5
CD163+(mono)/CD163+(poly)	19	0.13 ± 0.22	11.9 ± 20.2	15.3 ± 25.9
Scavenger Receptors				
CD68+	33	1.48 ± 1.71	135.8 ± 156.9	174.1 ± 201.2
CD36+	19	2.66 ± 2.62	244.0 ± 240.4	312.9 ± 308.2
Neutrophil-Derived Foam Cells				
CD66b+/CD68+	33	0.28 ± 0.73		32.9 ± 85.9
CD66b+/CD36+	29	0.14 ± 0.30		16.5 ± 35.3
Macrophage-Derived Foam Cells				
CD163+/CD68+	32	0.38 ± 1.21	34.9 ± 111.0	
**Neutrophil Elastase (NE)**	29	2.01 ± 5.78		236.5 ± 680.0
CD66b+/NE+	20	0.61 ± 1.42		71.8 ± 167.1
CD163 mono+/NE+	16	0.12 ± 0.18	11.0 ± 16.5	
CD66b+(mono)/CD163+(poly)				
All 26 plaques	26	0.17 ± 0.39	15.6 ± 35.8	20.0 ± 45.9
9/26 plaques - no co-localization	9	0.00 ± 0.00	0.0 ± 0.0	0.0 ± 0.0
14/26 plaques - low co-localization	14	0.07 ± 0.10	6.4 ± 9.2	8.2 ± 11.8
3/26 plaques - high co-localization	3	1.14 ± 0.50	77.3 ± 108.5 (§)	49.3 ± 35.9(§)
**3-Nitrotyrosin (3-NT)**	33	3.60 ± 4.12	330.3 ± 378.0	423.5 ± 484.7
3-NT/CD163+(poly)	16	0.63 ± 0.75	57.8 ± 68.8	74.1 ± 88.2
3-NT+/NE+	33	0.42 ± 1.03	38.5 ± 94.5	49.4 ± 121.2
Additional intra/extra-cellular markers				
HIF-1α	8	5.10 ± 2.69	467.9 ± 246.8	600.0 ± 316.5
VEGF	9	4.11 ± 7.72	377.1 ± 708.3	483.5 ± 908.2
CD31	14	1.37 ± 1.98	125.7 ± 181.7	161.2 ± 232.9
SMC actin	15	2.11 ± 1.76	193.6 ± 161.5	248.2 ± 207.1
**Lipids all**	27	15.49 ± 6.06	1421.1 ± 556.0	1822.4 ± 712.9
Intracellular lipids	27	3.28 ± 2.31	300.9± 211.9	385.9 ± 271.8
Extracellular lipids	27	9.59 ± 5.66	879.8 ± 519.3	1128.2 ± 665.9
Lipid crystals	27	4.05 ± 2.89	371.6 ± 265.1	476.5 ± 340.0
Collagen	33	23.14 ± 10.4	2122.9 ± 954.1	2722.5 ± 1223.5
Non-collagen proteins	33	6.53 ± 6.70	599.1 ± 614.7	768.2 ± 788.2

Data are reported as mean ± SD. (Poly), polyclonal antibody; (mono), monoclonal antibody. (*) Data are calculated as Relative % from the % stained area of CD163+ cells. (#) Data are calculated as Relative % from the % stained area of CD66b+ cells. (§) Data are calculated as relative % of average CD66b+ and CD163 expression from the high co-localization plaques.

**Table 2:** Pearson Correlation (R) analysis between % stained area of CD66b+ and CD163+ and % stained area of various markers and 3-nitrotyrosine (3-NT).

Correlations (% of stained area)	R	N	Significance (P-value) <
CD66b+ vs. extracellular lipids	-0.48	27	0.01
CD66b+ vs. all lipids	-0.42	27	0.03
CD163+ vs. intracellular lipids	0.44	27	0.02
CD163+ vs lipid crystals	-0.38	27	0.05
CD66b+ vs. CD68+	0.68	33	0.00001
CD66b+ vs. CD163+(mono)	0.76	32	0.00001
CD163+ vs. CD68+	0.68	32	0.00001
CD66b+/3-NT	0.34	33	0.05
CD163+(poly)/3-NT	0.46	29	0.01
CD163+(mono)/3-NT	0.37	32	0.03
CD66b+/CD163+ vs. 3-NT	0.65	26	0.0003
CD66b+/CD163+ vs. 163+/3-NT	0.55	16	0.03

N- number of plaques studied.

After overnight incubation with primary antibodues the slides were washed and incubated with 1/400 secondary antibodies in blocking buffer, at room temperature, for 40 min. Secondary antibodies included Cy2 (CF 488A)-conjugated goat anti-rabbit IgG and/or Cy5 (CF 647)-conjugated goat anti-mouse IgG (Biotium, Hayward, CA). Isotype controls included: purified mouse IgG1 (clone MOPC-21, BioLegend, San Diego, CA), and normal rabbit IgG (sc-2027, Santa Cruz Biotechnologies, Santa Cruz, CA). After 40 min. incubation, slides were washed and mounted with mounting medium containing 4’, 6-diamidino-2-phenylindole (DAPI) for nuclear staining (Vectashield H-1000, Vector lab. Inc. Burlingame, CA).

### Quantitative confocal fluorescence analysis

Slides were analyzed by a confocal laser scanning system (LSM 700) using Nikon E600 (Japan) fluorescence microscope and Plan Apo x 40 immersion oil objective. Fluorescent intensities were integrated with Image J software (Wayne Rasband, NIH, USA). Menders Overlap Coefficient (MOC) was used to quantify co-localization (17). Histological observations were recorded by an observer who was blinded to the clinical information.

### Statistical analysis

Continuous variables are presented as means and standard deviations (SD) and categorical variables are presented as absolute numbers and percentages. Pearson correlation analysis was performed to explore associations between variables. P < 0.05 was considered significant.

## Results

### Demographics, comorbidities, risk factors and medications of patients

**Table 3** presents the demographics, comorbidities, cardiovascular risk factors, and the use of medications for the 31 patients investigated. The average age of all patients was 71.9 ± 8.7 years, average BMI was 27 ± 3.7 Kg/m^2^. Male to female ratio was 2:1, and forty two percent of the patients were symptomatic, while most patients had at least one cardiovascular co-morbidity.

**Table 3:** Patients demographics, comorbidities, cardio-cerebrovascular risk factors and use of medication.

Variables	Values
Patients, N =	31
Age (years)	71.9 ± 8.7
BMI (Kg/m^2)^	27.7 ± 3.7
Gender	21M/10F
Comorbidities and risk factors - N (%)	
Symptomatic	13 (41.9)
Asymptomatic	18 (58.1)
IHD	10 (32.2)
sp/MI	8 (25.8)
CVA	16 (51.5)
TIA	7 (22.6)
HTN	27 (87.1)
PVD	13 (41.9)
DM	17 (54.8)
COPD	10 (32.2)
Hyperlipidemia	26 (83.9)
Smoking status - N (%)	
Current	10 (32.2)
Non-smoking	19 (61.3)
Past smoking	2 (6.4)
Drugs - N (%)	
Aspirin	21 (67.7)
Plavix	17 (54.8)
Clex	3 (9.7)
Statins	26 (83.9)
Ace inhibitors	20 (64.5)
Ca-channel blockers	15 (48.4)
β-blockers	13 (41.9)
α-blockers	5 (9.1)
Adrenergic α-agonist	3 (9.7)
Insulin	4 (12.9)
β-guanidine C cholesterol	18 (58.1)

Values are presented as average ± SD where appropriate. N - number of patients; in parentheses (%) percent of patients expressing the specific value. IHD, ischemic heart disease; s/p MI, status post myocardial infarction; CVA, stroke or cerebro-vascular accident; TIA, transient ischemic attack; HTN, hypertension; PVD, peripheral vascular disease; DM, diabetes mellitus; COPD, chronic obstructive pulmonary disease.

### Immunohistochemistry of plaques

**Figure 1:**
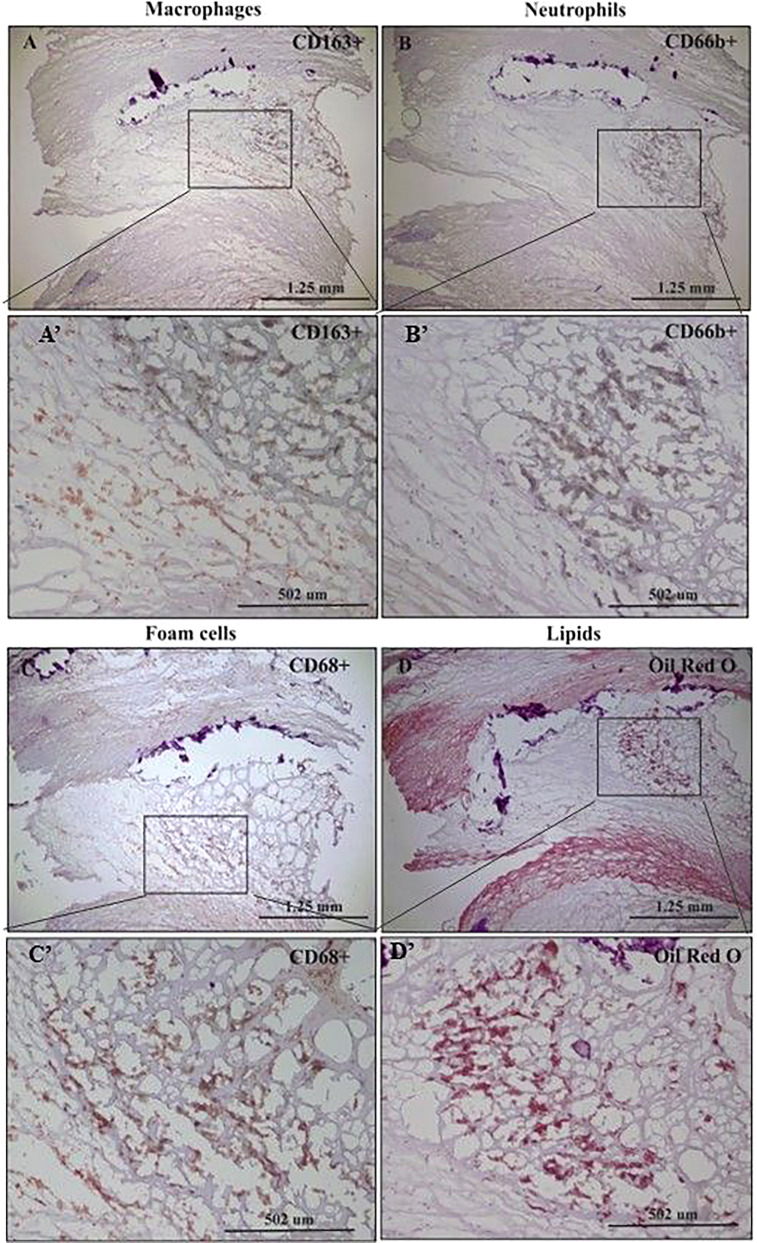
Immunohistochemical staining of macrophages (CD163+), neutrophils (CD66b+), foam cells (CD68+) and lipids in human carotid plaques. **(A)** Distribution of CD163-positive macrophages within area rich in foam cells, **(A’)** insertion of **(A)**. **(B)** Distribution of CD66b-positive neutrophils within area rich in foam cells, **(B’)** insertion of **(B)**. **(C)** Distribution of CD68-positive foam cells within the carotid plaque, **(C’)** insertion of **(C)**. **(D)** Distribution of lipids within area rich in foam cells, stained with Oil Red O, **(D’)** insertion of **(D)**.

**Figure 2:**
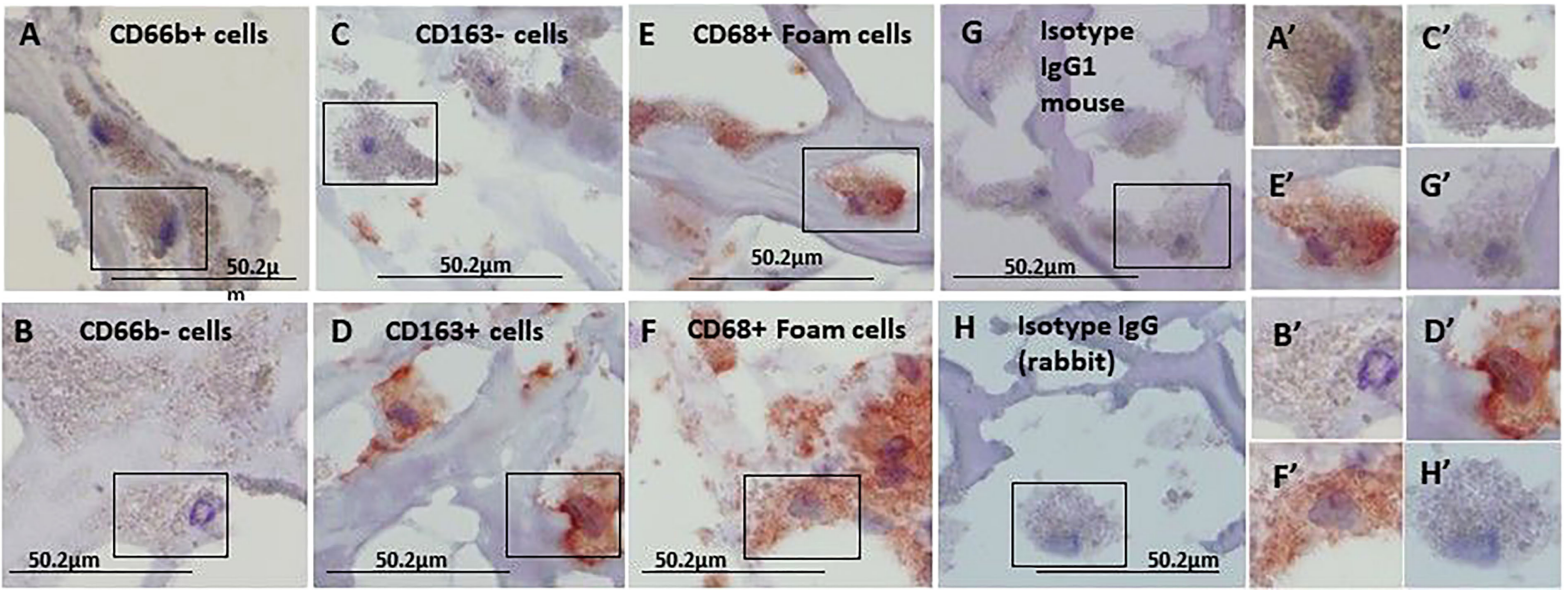
Immunohistochemical staining for CD66b neutrophils, CD163 macrophages and CD68 foam cells, each stained in a consecutive section of the carotid plaques. **(A)** a CD66b-positive cell and **(B)** a CD66b-negative cell are both from the same section stained with anti-CD66b antibody. **(C)** a CD163-negative and **(D)** CD163-positive cell, both are from the same section stained with anti-CD163 antibody. In **(E, F)** CD68-positive cells were stained with anti-CD68 antibody. **(G)** Isotype control IgG1 for CD66b and CD163. In **(H)** Isotype control IgG (rabbit) for CD68. **(A’–H’)** cell magnifications corresponding to **(A–H)**. These sections were taken from a plaque negative for CD66b/CD163 co-localization, as determined by immunofluorescence with confocal microscope. Original magnification: A- H x100, scale bar: 50.2 µm, insert: 2x100, scale bar: 50.2 µm.

**Figure 3:**
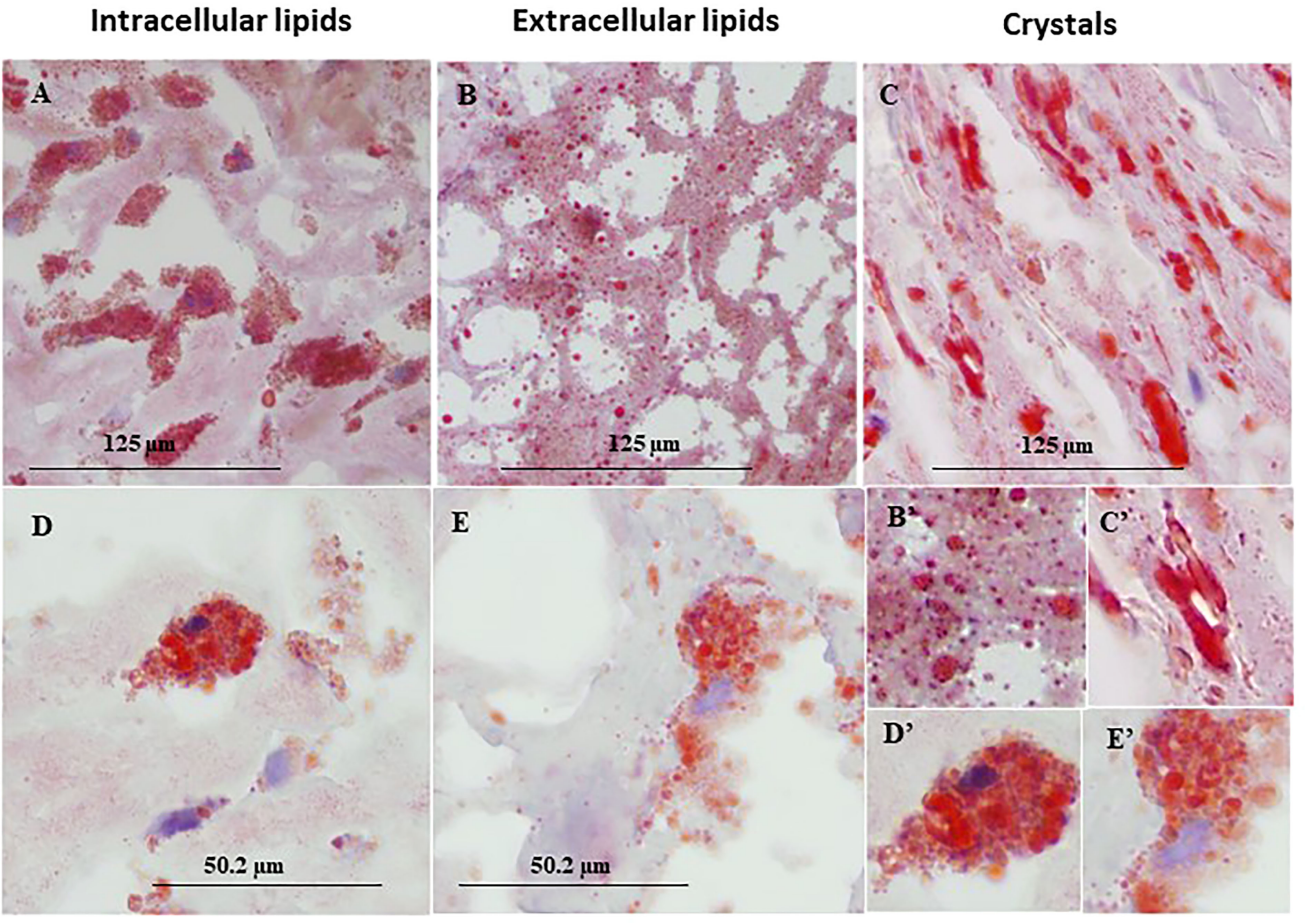
Representative immunohistochemical lipid deposits stained by Oil Red O in lipid rich area of a human carotid plaque. **(A)** intracellular lipids, **(B)** extracellular lipids, **(C)** lipid crystals, **(D, E)** foam cells prevalent in human carotid plaques, both taken from the same plaque section. **(B'–E')** enlargements corresponding to **(B–E)**. Original magnification: **(A–C)**, x40, scale bar: 125 µm; **(D, E)**, x100, scale bar: 50.2 µm; inserts: 2x100, scale bar: 50.2 µm.

**Figure 1** represents immunohistochemical staining of a carotid plaque for macrophages, neutrophils, foam cells, and lipids. The immuno-histological staining experiments were performed for each marker alone in a separate consecutive section, the areas depicted for CD163+, CD66b+, CD68+, and Oil Red O staining (A- D), are similar for each sections. This plaque is a representative of one of the plaques that were negative for CD66b/CD163 co-localization. It is evident from these sections that most of the CD163+ stained macrophages (A, A’) and CD66b+ neutrophils (B, B’) accumulated in areas rich in CD68+ foam cells (C, C’) and lipids (D, D’). Both monocytes/macrophages and neutrophils are predominantly present in CD68 positive areas which are also lipid rich areas in the carotid plaque. Detailed immunohistochemical analysis for CD66b, CD163 and CD68 staining under a higher magnification is depicted in **Figure 2**. The photomicrographs in **Figure 2** represent one of the plaques that was negative for CD66b/CD163 co-localization. Typical cells in a section stained for CD66b show a CD66b+ cell (A) and a CD66b-negative cell in (B). Representative cells from a section stained for CD163 show a CD163-negative cell in (C) and a CD163-positive cell in (D). Representative CD68+ stained cells from a CD68 stained section in (E, F). Isotype controls were negative for all markers (G, H). **Figures 2A’–H’** depict magnifications of the various cell markers which correspond to (A-H). Of note, the CD66b+ cells presented in this Figure are larger in size than classical circulating blood neutrophils which are about 12-15 µm in diameter, and do not have a multi-lobed nucleus. **Figures 3A–C** depict the distribution of intracellular and extracellular lipids stained with Oil Red O, as well as magnifications of typical lipid laden cells in (3D, E) and (3D’, E’).

### Immunofluorescence and co-localizations of neutrophils and macrophages markers

**Figure 4:**
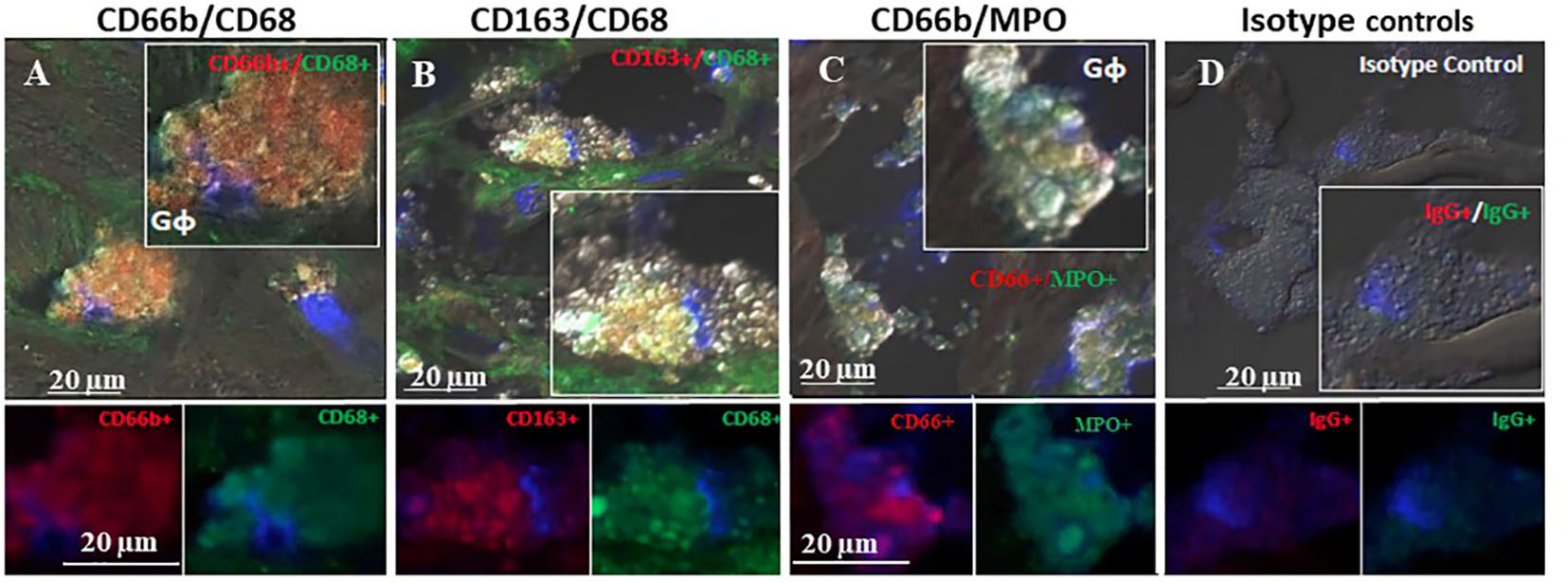
Representative double immunofluorescence staining for CD66b, CD163, CD68, and MPO in human carotid plaque sections. **(A)** co-expression of CD66b (red) in neutrophils co-expressing CD68 (green) scavenger receptors, **(B)** co-expression of CD163 in macrophages (red) co-expressing CD68 (green) scavenger receptors, **(C)** co-expression of CD66b (red) in neutrophils co-expressing MPO (green), **(D)** isotype control staining for mouse IgG1 and rabbit IgG followed by CF647 goat anti-mouse IgG (red) and CF488 goat anti-rabbit IgG (green).Top photomicrographs **(A–D)** represent merged channels of co-expression and enlarged inserts. Below enlarged inserts represent each channel (red or green) separately. Scale bar: 20 µm.

**Figure 5:**
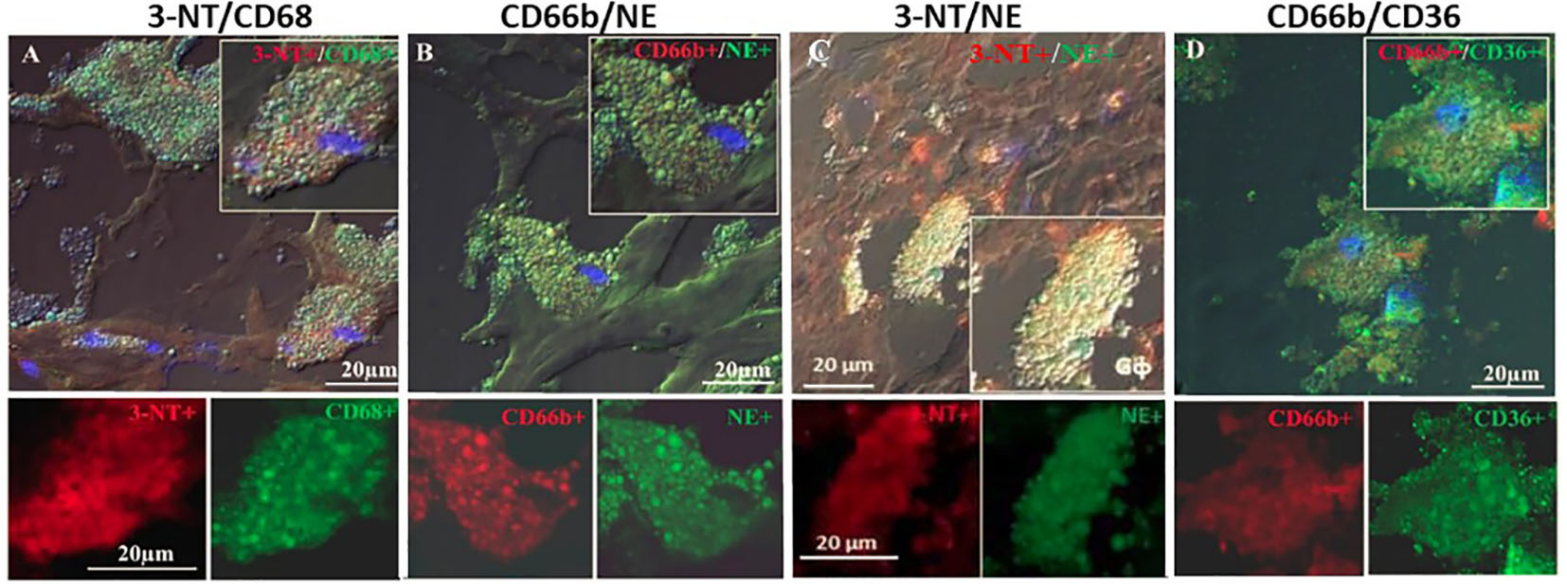
Representative double immunofluorescence staining for 3-NT, CD68, CD66b, NE and CD36 in human carotid plaque sections. **(A)** co-expression of 3-NT (red) and CD68 (green), **(B)** co-expression of CD66b neutrophils and NE (green), **(C)** co-expression of 3-NT (red) and NE (green), **(D)** co-expression of CD66b neutrophil (red) and CD36 scavenger receptor (green). Isotype control staining for mouse IgG and rabbit IgG followed by CF647 goat anti-mouse IgG (red) and CF488 goat anti-rabbit IgG (green), as shown in **Figure 4D**. Top photomicrographs **(A–D)** represent merged channels of co-expression and enlarged inserts. Below enlarged inserts represent each channel (red or green) separately. Scale bar: 20 µm.

**Figure 4** represents immunofluorescence of cells analyzed by confocal microscopy. Neutrophils were identified by CD66b+ expression and co-localized with CD68+ (A), macrophages were identified by CD163+ expression and co-localized with CD68+ (B), both cell types co-expressed the scavenger receptor CD68. Plaque CD66b+ neutrophils also co-localized with MPO (C). Isotype controls for monoclonal (red) and polyclonal (green) antibodies were negative (D). All in all, the large neutrophilic cells are very similar to the macrophages-foam cells in size and morphology but differ in the specific markers. Double staining by immunofluorescence revealed that out of 33 plaques analyzed, 31 plaques expressed the specific CD66b neutrophil marker and 21 of the plaques co-expressed CD66b+/CD68+. Thirty-two plaques were CD163 positive for macrophage-foam cells and 22 plaques co-expressed CD163+/CD68+. Plaque cells and CD66b-positive cells also co-localized with additional markers as depicted in **Figure 5** including: 3-NT+/CD68+ (A), CD66b+/NE+ (B), 3-NT+/NE+ (C) and CD66b+/CD36+ (D). Isotype controls (for mouse monoclonal and rabbit polyclonal antibodies) were negative for the corresponding markers (same as in **Figure 4D**).

### Quantitative analysis of plaque markers

**Table 1** presents quantitative analyses of the markers investigated in these plaques including cellular, inflammatory, and oxidative stress markers. The data are presented by the mean percent of the stained area of at least 3 different sections from the center of each plaque for each single or double stained markers. In each section at least five different fields were analyzed for each of the various markers determined. Co-expression was determined in the same section, but for determination of each set of different co-localization markers, consecutive sections were used. The absolute % stained area of the cells in the plaque was also converted to relative percentage of the cells. The expression of CD163+ and that of CD66b+ were considered as 100% each, and all values were calculated as relative percentages. These calculations exemplify the relatively small % of the area that was stained by the cells as compared to other components of the plaque such as lipids, vessels, endothelial cells smooth muscle cells collagens and non-collagen proteins etc. For instance, collagen and non-collagen proteins are more than 25-fold higher as relative to CD66b+ or CD163+ expression. The percent area of overlap between CD66b+ and NE+ was more than 70% by co-localization by double-immunostaining analysis. Thirty tree percent of CD66b+ cells co-localized with CD68+, and 16.5% co-localized with CD36+. The percent area expressed by CD163+ macrophages, using both monoclonal (mono) or polyclonal (poly) antibodies, was similar or higher (respectively) than that of CD66b+ expressing cells, but this difference was not statistically significant. The percent area of overlap between CD163+ macrophages and CD68+ was 35% by co-localization by double-immunostaining analysis. Also, macrophage co-localization of CD163mono (red) and CD163poly antibodies (green), was relatively low, as compared to the area expressed by each antibody alone, indicating that each of the antibodies recognized a different epitope.

### Co-localization of neutrophils/macrophages markers in plaques

In order to clarify whether CD66b+ neutrophils and CD163+ macrophages are distinct populations of cells, CD66b (mono)/CD163(poly) co-localization was performed. Co-localization of CD66b/CD163 was quantified in 26 of the plaques and largely varied between the samples (**Table 1** **).** In nine of the plaques there was no CD66b/CD163 co-localization at all. In 14 plaques co-localization was low and only in three plaques CD66b+/CD163+ co-localization was high. These three patients’ plaques exceeded the group’s average by at least one standard deviation (SD). There was a 15-20% in CD66b+/CD163+ co-localization across all plaques as compared to the relative percent of each of the CD66b+ or CD163+ cells. While in 9 plaques there was no co-localization, in the plaques with low CD66b+/CD163+ co-localization, 8 -12% of the cells co-localized as compared to CD66b+ or CD163+. However, in the 3 plaques with the high co-localization the amounts of both CD163+ and CD66b+ expressing cells were much higher than the average values for all other plaques. Thus, the CD66b+/CD163+ co-localization was calculated separately for these plaques, using their average CD66b+ or CD163+ values of expression as a reference. A 50% to about 80% co-localization was found for CD66b+ and CD163+ cells, respectively (in **Table 1**).

**Figure 6:**
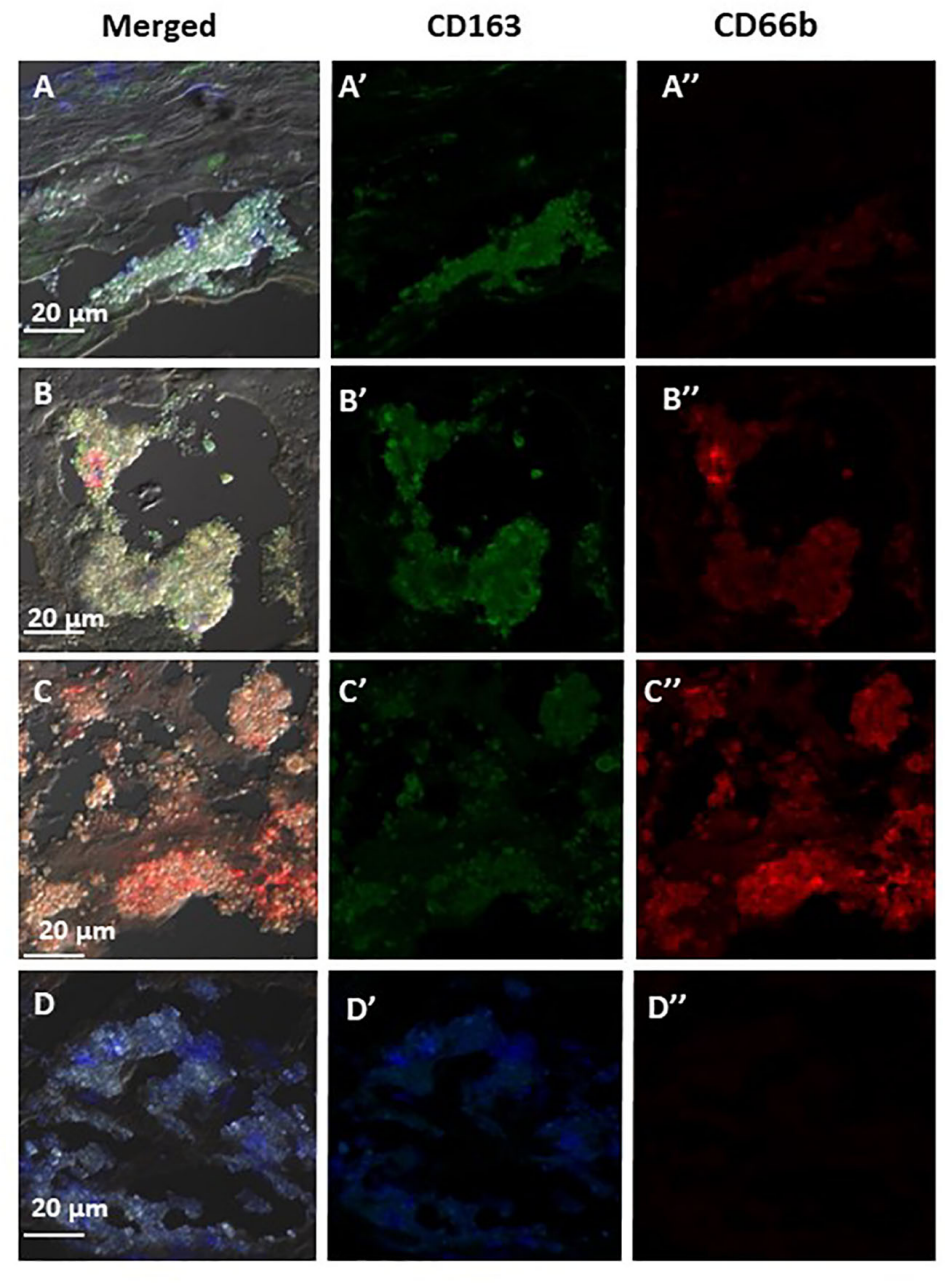
Representative immunofluorescence by double staining of CD66b/CD163, depicting typical co-localizations of various carotid plaques. In **(A)** merged co-expression of CD66b+/CD163+, in **(A’)** green channel, higher expression of CD163+ as compared to **(A’’)** red channel for CD66b. In **(B)** merged CD66b+/CD163+ co-expression with similar intensities of expression for CD163+ **(B’)** and CD66b+ **(B’’).** In **(C)** merged CD66b+/CD163+co-expression, in **(C’)** low CD163+ expression, in **(C’’)** higher expression of CD66b+ as compared to CD163. In **(D)** isotype controls for rabbit polyclonal antibody **(D’)** and for mouse monoclonal antibody **(D’’)**, as specified in **Figure 4D**.

**Figure 6** represents three of the CD66b+/CD163+ types of co-localization visualized. In (A) CD163+ macrophage expression was higher than that of CD66b+ (A’, A”). In (B) the expression is about equal in both cell types (B’, B”) and in (C) there is a stronger expression by CD66b+ neutrophils (C”) as compared to CD163+ macrophages (C’). In (D) isotope control is presented for all. In plaques with a low co-localization, the expressions for both markers were lower and fainter, but the same types of co-expression patterns were noted as depicted in **Figures 6A–C**. In some plaques there was overall a CD66b+ dominance over CD163+, in other plaques CD163+ expression was higher or it was about the same. Yet, all types of co-expressing cells represented in **Figure 6** were present in different fields of the plaques, with lower intensities.

### Correlation analyses

Correlation analysis (**Table 2** **)** revealed that the CD66b+ expressing cells were significantly negatively correlated with extracellular lipids and with all lipids (extra/intracellular and crystals), whereas CD163+ cells were significantly positively correlated with intracellular lipids and negatively with lipid crystals. Also, CD66b+ cells were highly significantly positively correlated with CD68 and with CD163+ expressing cells, and CD163+ cells were also highly significantly correlated with CD68+. Both CD163+ macrophages and CD66b+ neutrophils were positively correlated with the nitrosative stress marker 3-nitrotyrosine (3-NT). Of note, neutrophils/macrophages co-expressing CD66b+/CD163+ were also highly significantly correlated with 3-NT. Moreover, CD66b+/CD163+ also correlated with a macrophage subgroup co-expressing CD163+/3-NT+.

### Hypoxia associated markers

**Figure 7:**
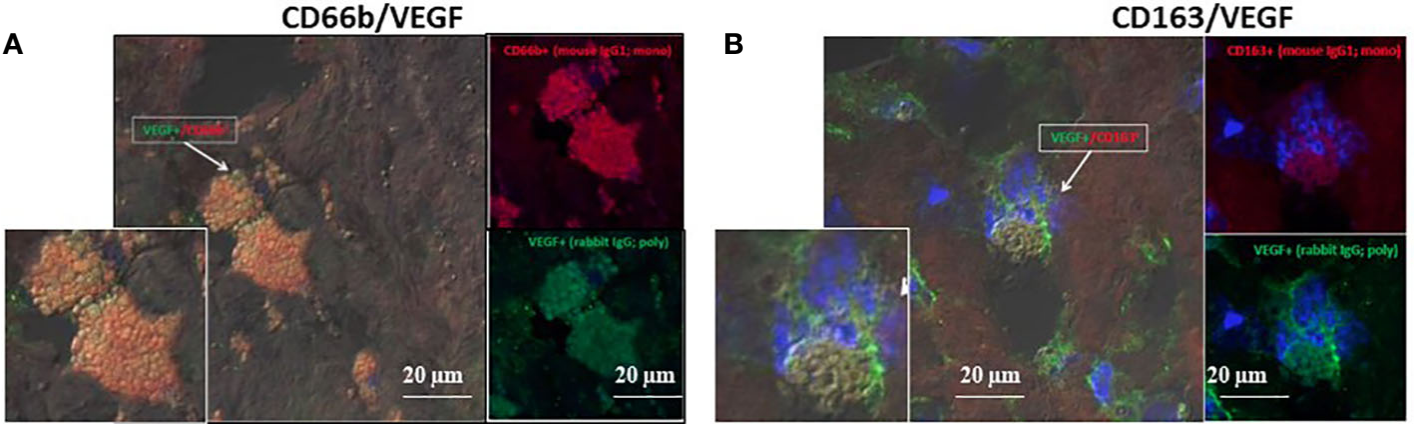
VEGF co-expression by double immunofluorescence staining for CD66b neutrophils and CD163 macrophages in human carotid plaques. **(A)** co-expression of CD66b-positive neutrophils (red) and VEGF (green), **(B)** co-expression of CD163-positive macrophages (red) and VEGF (green). Isotype control staining for mouse IgG and rabbit IgG followed by CF647 goat anti-mouse IgG (red) and CF488 goat anti-rabbit IgG (green), as shown in **Figure 4D**. **(A, B)** represent merged co-expression. In right expression for each stain separately and enlarged inserts on the left.

The developing plaques are characterized by hypoxic stress, therefore HIF-1α and VEGF were quantitatively determined since both can facilitate vessel growth. CD163+ and CD66b+ cells expressed HIF-1α (data not shown) and VEGF. **Figure 7** represents co-expression of CD66b+ neutrophils and CD163+ macrophages with VEGF, the downstream gene of HIF-1α. Additionally, the presence of endothelial CD31 and SMC-actin representing vascular remodeling was also confirmed in the plaques investigated as depicted in **Table 1**.

**Table 4** summarizes all the markers studied including: for macrophages and neutrophils, various co-localization markers for both types of cells, and additional cellular markers stained by immunofluorescence.

**Table 4:** A list of the various CD and cellular markers studied, and the number of plaques quantified by confocal microscopy.

Expression of single markers - N	Neutrophils and co-expression of markers-N	Macropages and co-expression of markers -N
CD68 - 33 CD36 - 19 NE - 29 3-NT - 33 HIF-1α - 8 VEGF - 9 CD31 -14 SMC-actin - 15 Lipids - 27	CD66b - 33 CD66b/CD68 - 33 CD66b/CD36 - 29 CD66b/CD163(poly) - 26 CD66b/NE - 20 3-NT/NE - 33	CD163(mono) - 32 CD163 (poly) -29 CD163(mono)/CD163 (poly) - 19 CD163(momo)/CD68 - 31 CD163(momo)/NE - 16 3-NT/CD163(poly) - 16

Letters in RED represent mouse monoclonal antibodies. Letters in GREEN represent rabbit polyclonal antibodies. N, represents the number of carotid plaques quantified for each of the markers. (Mono), monoclonal antibody; (poly), polyclonal antibody.

## Discussion

It is commonly known that neutrophils are short-lived in *ex vivo* conditions (18). Yet, in earlier studies we described the spontaneous development of a small sub-population of neutrophil-derived Gφ with extended life span in culture (2, 3). We were intrigued by their vastly enlarged size observed *in vitro* after a few days in culture. Thus, we divided fresh neutrophils into two sub-groups, each labeled with a different PKH stain (green or red), mixed them together and followed their fate daily, microscopically. As expected, most of the neutrophils died within 24 hrs., however, the few neutrophils that remained viable (0.01- 0.02%), avidly internalized remnants of dead neutrophils and cellular debris. Subsequently, transforming within 3-5 days in culture from classical small-sized neutrophils with a multi-lobed nucleus into large phagocytic cells with a rounded nucleus. They also internalized oxidized LDL, zymosan, and latex upon stimulation, and generated ROS. These newly developed neutrophils resembled macrophage-foam cells morphologically but expressed neutrophil specific markers (CD66b, CD63, NE, MPO), and were negative for monocytic or dendritic markers. Importantly, FACS analysis revealed that the cultures consisted of pure neutrophils. Moreover, by mixing and co-culturing autologous neutrophils and monocytes, fewer Gφ developed in culture.

In the current study we sought to investigate the potential relevance of Gφ to inflammatory-atherosclerotic conditions ***in vivo***, using the human carotid plaque as a model. Several important findings emerged from this study. **First,** the presence of CD66b+ neutrophils and 163+ macrophages was observed in lipid-rich areas of the plaque. **Second**, Both CD66b+ and CD163+ expressing cells also co-expressed CD68 scavenger receptors and both had similar morphology, size, and lipid content which is characteristic of foam cells. **Third,** CD66b+/CD163+ neutrophil/macrophage co-localization was also noted. This co-expression varied widely between the plaques, ranging from zero in some of the plaques, to low in most plaques. A high CD66b+/CD163+ co-localization was also found in few plaques. **Fourth,** the CD66b+ cells in the plaques also co-expressed additional specific neutrophil markers visualized by confocal immunofluorescence double staining. They co-localized with NE, MPO, and CD63 (data not sown), as well as with CD36, which is another scavenger receptor that was previously shown to co-localize with CD66b+ cultured Gφ. **Fifth,** CD66b+ neutrophils and CD163+ macrophages co-expressed oxidative-nitrosative stress activity detected by co-localization of 3-NT+/NE+ and 3-NT+/CD163+ cells, respectively. **Sixth,** the presence of VEGF was demonstrated by neutrophils co-expressing CD66b+/VEGF+ and macrophages co-expressing CD163+/VEGF.

We hypothesize that CD66b+ cells which did not co-localize with CD163+, resemble the Gφ which developed in pure neutrophil cultures and represent a type of an inflammatory/anti-inflammatory-activated neutrophil phenotype. Yet, other neutrophils that co-expressed CD66b+/CD163+ to varying degrees, exhibit neutrophil/macrophage plasticity and trans-differentiation to monocytes/macrophages or monocytes/macrophages hybrids. Evidently, these hybrid cells might have combined neutrophil-macrophage properties. The latter co-localization findings, in particularly, demonstrate the complexity and the difficulties in studying intricate tissues such as carotid plaques, and likely each plaque represents a different stage in development and severity.

The neutrophil is the most abundant white blood cell in the circulation, yet, it received little attention in the pathophysiology of atherosclerosis (19, 20). However, in the last decade the evidence is gradually mounting as to its involvement and significance in various stages of plaque formation, development, and rupture (18, 21). In earlier studies, CD66b+ neutrophils’ localization within plaques, and their numbers were strongly associated with histopathologic features of rupture-prone plaques, implicating their involvement in plaque destabilization (10). A massive neutrophil infiltration was found in culprit lesions of autopsy specimens from acute coronary syndrome (ACS) patients as compared to the rare neutrophil infiltration in patients who died from non-cardiovascular disease (12). Also, in seven severely atherosclerotic vulnerable plaques investigated, infiltrating neutrophils appeared activated and were generating oxidants (11).

More recent studies, however, demonstrated that neutrophils accelerate all stages of atherosclerotic plaque development including plaque initiation, progression, instability, and rupture, staring with the onset of atherosclerosis through damage to the arterial wall caused by oscillating shear forces, hyperlipidemia, and inflammatory cytokines. These promote endothelial cell activation and adhesion by myeloid cells, consequently infiltrating into the arterial intima. The accumulation of immune cells, lipoproteins and cell debris in the arterial intima contribute to atherosclerotic plaque development and instability, promoting plaque rupture and cardiovascular complications (21). Specifically, neutrophils were shown to be the first to extravasate to sites of inflammation and to recruit and regulate monocyte entry into atherosclerotic lesions, thereby accelerating foam cell formation (22). Neutrophils and their secretory products also affected macrophage fate and function (21, 23), and were also shown to accumulate in injured areas of the plaque and to surround damaged vasculature, promoting tissue repair by inducing angiogenesis and revascularization (24, 25). Collectively, these studies clearly demonstrate the significance of CD66b+ neutrophils in all stages of plaque development and tissue repair.

Unlike the studies reported thus far, implicating neutrophils in various stages of plaque development, we focused our attention on identifying the presence of neutrophils resembling the Gφ that were previously reported to develop spontaneously in culture conditions *in vitro*, by characterizing their morphology and the markers co-expressed in plaque neutrophils.

In the current study, neutrophils expressing CD66b+/NE+/MPO+ were shown to reside in lipid-rich areas of the plaques as were CD163+ foam cell macrophages, and both cell types co-expressed the scavenger receptors CD68 and CD36. Nonetheless, in some of the plaques also varying sums of CD66b+/CD163+ co-localization was noted. However, CD66b+ cells looked like macrophages-foam cells morphologically and expressed CD68 and CD36 scavenger receptors which are considered exclusive as macrophages markers. Thus, the co-localization of both scavenger receptors - CD68 and CD36 with CD66b+ expressing cells might have facilitated their transformation into foam cells, similarly to Gφ in culture. It is therefore likely that the rooted belief that CD68 is exclusively expressed by cells of the monocytic lineage and macrophage-foam cells, lead some investigations in earlier studies to identify plaque macrophages based solely on the expression of CD68, consequently, ignoring the possibility that other cell types present in plaques might express similar phenotypes (26, 27). This approach might have resulted in underestimation of the contribution of neutrophils to plaque pathology (27).

To clarify whether CD66b+ and CD163+ cells in carotid plaques are distinct cell populations, CD66b/CD163 co-localization was performed. Substantial differences were noted between plaques. In a third, co-localization was negative, suggesting that CD66b+ and CD163+ represent distinct cell populations. In other plaques, co-localization varied, but was mostly low, whilst, in few plaques it was highly positive. Hence, indicating that CD66b+/CD163+ co-localization is a gradual process of neutrophil to macrophage (or vice versa) trans-differentiation. Evidently, neutrophil plasticity and trans-differentiation are increasingly being recognized. Neutrophils were shown to express features of other myeloid cells including eosinophils, antigen presenting cells (APCs), dendritic cells (DCs) as well as monocytes (13). Trans-differentiation of neutrophils to APCs was found at inflammatory sites of autoimmune arthritis (28). Long-lived neutrophils with phenotypic plasticity, trans-differentiated into neutrophil-dendritic cells hybrids (N-DCs), expressing properties of neutrophils and DCs, accompanied with a potent anti-fungal activity (29). In rheumatoid arthritis, N-DCs were found concurrently with CD66b+ neutrophils co-expressing features of APCs. Moreover, increased ROS generation in the synovial fluid of these patients promoted trans-differentiation of neutrophils to N-DCs (30). Also, mouse bone marrow resident cells with neutrophilic phenotype were able to differentiate to monocytes when cultured with M-CSF (31). Taken together these recent findings support a high neutrophil plasticity and trans-differentiation. Yet, we cannot rule out the possibilities that there is a macrophage to neutrophil trans-differentiation or that CD66b+/CD163+ co-expressing cells in the carotid plaques might have resulted from macrophages phagocytosing dead neutrophils and adopting their markers.

Of note, in carotid plaques, smooth muscle cells (SMCs) were shown to trans-differentiate and acquire macrophage-like phenotype, contributing to atherosclerotic plaque pathogenesis due to their phenotypic plasticity and the ability to mimic macrophages. This trait resulted in underestimating SMCs’ involvement in plaque pathology (32, 33). Moreover, these latter findings support a fundamental mechanism of myeloid cells (and neutrophils in particularly), in which they rapidly adapt to their immediate tissue environment or to diverse biological microenvironmental stimuli in health and disease through plasticity and trans-differentiation (13).

Correlation analysis revealed that CD66b+ and CD163+ cells were significantly positively correlated with CD68. While CD66b+ cells were negatively correlated with extracellular lipid and all lipids, CD163+ cells were significantly positively correlated with intracellular lipids, hence becoming foam cells. Given that CD66b expression in neutrophils is positively correlated with the expression of CD68, and negatively with all lipids indicates that also CD66b+ might become foam cells in lipid rich areas of the plaques, and thus, represent a supportive role by acting as a backup system when the macrophage clearing system is insufficient and/or overwhelmed (34). For instance, by eliminating and protecting the arterial wall from excessive lipid accumulation.

Among other factors, increased oxidant activity in plaques is associated with endothelial dysfunction (19) and neutrophil trans-differentiation into N-DCs (30). Macrophages co-expressed 3-NT+/CD163+ while co-expression in neutrophils was detected in 3-NT+/NE+ cells. Both cell types were significantly positively correlated with quantitative expression of 3-NT, indicating that in carotid plaques, nitrosative stress is associated with both. Interestingly, cells co-expressing CD66b+/CD163+ were highly positively correlated with 3-NT, as were neutrophils that trans-differentiated into N-DCs (30). Activated macrophages are known to produce NO through iNOS. However, the data are scarce for neutrophils. Yet, few studies show that all three NOS isoforms are present and generate NO in adult human neutrophils (35). Moreover, MPO, a major factor in oxidizing products in neutrophils was shown to produce nitrating oxidants *in vivo* (36). In addition, 3-NT levels were shown to decrease in stable plaques and increase in unstable plaques (37). In a study identifying CD68+ cells as representing only macrophages, carotid 3-NT and NOSII co-localized in late stages of atherosclerosis, suggesting that NOS expression is involved in oxidation/peroxidation of lipids promoting carotid formation (27). Hence, 3-NT+/NE+ neutrophils in carotid plaques can contribute to free radicals by oxidizing/peroxidizing lipids, and thus, facilitate foam cell formation through scavenger receptor regulation.

A great number of cytokines and chemokines were identified over the last few years in neutrophils, signifying their involvement in inflammatory and immune processes as well as in angiogenesis and repair (38). In earlier studies, neutrophils were shown to synthesize and store molecules with angiogenic activity including VEGF (39), which participates in angiogenesis and neovascularization, to promote plaque destabilization and thrombeolitic events (40). However, VEGF can have dual effects, detrimental as well as beneficial, by protecting endothelial cells (41). Besides VEGF, intraplaque neutrophils were also shown to produce IL-8 and elastase which are all crucial for the development and progression of plaques (42). Herein, we confirmed that both CD163+ and CD66b+ cells in plaques co-expressed VEGF, as illustrated in **Figure 7**.

The limitations of this study should be acknowledged. In the immuno-histological staining experiments, we could not perform co-localization with two antibodies simultaneously due to limitations in the kit’s procedure. However, consecutive sections were analyzed basically at the same area of the plaque (see **Figure 1**). Immunofluorescence and co-localization experiments with confocal microscopy, also compensated to some extent for this limitation.

The number of plaques investigated was relatively small. However, it should also be acknowledged that these experimental procedures for identifying the markers investigated on a large-scale study are tedious, time consuming and very expensive. Moreover, unlike tissue culture studies or studies with inbred animal strains, each plaque is a complex tissue, with a unique morphology and cellular composition which was endarterectomized at different stages of its development and exposed to different environmental/microenvironmental signals. Undoubtedly, large-scale studies are needed to further clarify the characteristics and functions of CD66b+ and CD66b/CD163 co-expressing cells and their contribution to plaque pathology. It is still an open question – weather it is neutrophil to macrophage trans-differentiation or vice versa, or do they represent neutrophil-macrophage hybrids? Or possibly CD66b+/CD163+ cells are macrophages phagocytosing dead neutrophils and adopting their markers? However, since neutrophils are the first to arrive to plaques, and to facilitate many monocyte functions they might be the ones that respond swiftly to signals and acquire macrophage markers.

## Conclusions

In surveying the literature, we could not find a report demonstrating CD66b+ neutrophils resembling the ones reported herein. Neutrophils that are large foam cells with a rounded nucleus but positive for NE/MPO/CD63, for the non-neutrophil markers, CD68 and CD36 scavenger receptors, and concurrent neutrophil-macrophage CD66b+/CD163+ co-expression to varying extents in many of the plaques. Some of the earlier studies presenting photomicrographs of CD66b+ positive cells in human carotid plaques specimens were stained only by immunohistochemistry, validating the presence of neutrophils solely based on their specific CD66b+ markers. In these studies, the resolutions presented for CD66b+ cells were illustrated by very small magnifications, or by molecular methods, making it impossible to appreciate their size or morphology (10–12, 38, 42–44) as described herein. The CD66b+/CD163+ cells’ co-localizations depicted here might represent a neutrophil-macrophage hybrid cell or trans-differentiation of one type of cell to the other. Since CD66b+ cells and lipids were significantly inversely correlated, we hypothesize that the presence of CD66b+ cells in lipid-rich areas might represent a supportive role in helping macrophage foam-cells to combat inflammation and eliminate and protect the arterial wall from excessive lipid accumulation. On the other hand, 3-NT which promotes oxidation of lipids, could facilitate carotid plaque formation. Of note, identifying these unique subpopulations of neutrophils in plaques and in additional *in vivo* inflammatory conditions may provide valuable insights into the regulation of inflammatory responses during the initiation and progression of atherosclerotic plaque development. Moreover, the *in vitro* studies described earlier can be utilized to further facilitate the understanding of inflammatory/atherosclerotic neutrophil biology.

## Data availability statement

The original contributions presented in the study are included in the article/supplementary material. Further inquiries can be directed to the corresponding author.

## Ethics statement

The studies involving human participants were reviewed and approved by The Local Humans Rights Committee of RAMBAM Medical Center (RMB-0175-14), according to the declaration of Helsinki. The patients/participants provided their written informed consent to participate in this study.

## Author contributions

LL led the project. LL and AH participated in the conceptual framework of the project. AH recruited the patients. AH and ES-o performed endarterectomy. ES-o collected and organized patients’ clinical data. LL analyzed and interpreted the data, wrote and edited the manuscript and edited the proof. All authors contributed to the article and approved the submitted version.
